# Wettability and Functionality of Extruded Potato Starch Films Enriched with Edible Oil

**DOI:** 10.3390/molecules31142547

**Published:** 2026-07-22

**Authors:** Marzena Włodarczyk-Stasiak, Małgorzata Jurak, Agnieszka Ewa Wiącek

**Affiliations:** 1 Department of Analysis and Evaluation of Food Quality, Faculty of Food Science and Biotechnology, University of Life Sciences in Lublin, Skromna Street 8, 20-704 Lublin, Poland; 2 Department of Interfacial Phenomena, Institute of Chemical Sciences, Faculty of Chemistry, Maria Curie-Skłodowska University, Maria Curie-Skłodowska Sq. 3, 20-031 Lublin, Poland; malgorzata.jurak@mail.umcs.pl

**Keywords:** extruded potato starch, FTIR, profilometric images, contact angle measurements, surface free energy

## Abstract

Potato starch extrudates, chemically modified by a K_2_CO_3_ catalyst and enriched with two types of edible oils (rapeseed or sunflower) at varying concentrations (3%, 6%, 9%), were used as film substrates. This study was carried out as a continuation of previous research on analogous extrudates in the form of dry powders and liquid solutions. The main objective was to determine surface properties of polysaccharide films as a function of oil type and concentration to monitor their wettability, biocompatibility, and functional characteristics (e.g., transparency, colour, thickness, flexibility, solubility). Advancing and receding contact angles for polar liquids (water and formamide) and non-polar diiodomethane were measured on the base and oil-modified extruded starch films. Based on these measurements, the surface free energy of the films was determined using the contact angle hysteresis (CAH) model. Optical profilometry confirmed the wettability results through surface morphology and roughness evaluation. Additionally, FTIR analysis of the films was compared to the FTIR spectra of extruded starch powders. Combining these methods provided an in-depth characterization of the films, thereby improving control over their stability and wettability, which is essential for applications in the pharmaceutical and food industries to extend product freshness and enhance resistance to oxidation and spoilage.

## 1. Introduction

Most food products reaching consumers are packaged; therefore, packaging constitutes an integral component of such products. The primary purpose of packaging is to extend product shelf life, but it also contributes to product attractiveness. Nowadays, consumers are increasingly environmentally aware, which is why conventional plastic packaging is progressively being replaced with biodegradable and environmentally friendly materials. The large volume of packaging waste accumulating in landfills and the difficulties associated with its disposal have driven growing interest in alternative solutions, such as edible films, coatings, and foils derived from natural raw materials [1,2,3,4].

The main materials used to produce edible packaging include proteins, polysaccharides, and lipids [5,6,7]. One of the most commonly used polysaccharides for film and coating production is starch, a readily available and inexpensive biopolymer with film-forming properties. Native starch does not always meet the requirements expected of packaging materials. Therefore, to improve its functionality, it is subjected to targeted modifications aimed at enhancing its mechanical and barrier properties [8]. Starch films and coatings can protect food against moisture loss, as well as against the penetration of oxygen and carbon dioxide [9,10], and against the effects of adverse environmental and microbiological factors [11]. Lipid-containing films have been shown to have promising effects on preserving the quality of fresh fruit [12], protecting colour [13], and extending the freshness of sliced fruit [14,15,16].

Based on literature data [8,17,18], the combination of starch and vegetable oils is a promising direction in the development of edible films to extend food shelf life; however, there is limited data on their effectiveness and in-depth physicochemical characterization. Therefore, one of the main goals of this paper was to expand the characterization of the films for food applications. These films were made from starch extrudates modified with two types of edible oils, the powders and aqueous solutions of which, were discussed in detail in our earlier papers in this series [19,20].

Additionally, edible films or, in a broader sense, edible packaging can improve sensory and visual attractiveness through the incorporation of functional substances such as colourants, flavourings, and antioxidants, while the addition of minerals or vitamins can enhance the nutritional and health value of the product [9,17,21]. The choice of rapeseed and sunflower oil for film-forming systems was dictated by their favourable balance of omega-6 to omega-3 fatty acids, health safety, and their thermal stability during extrusion. Another health benefit of oil addition can encompass changing the ratio of rapidly digestible starch to resistant starch. For instance, Nisitthichai et al. [21] demonstrated that a 10% addition of palm or coconut oil reduced the amount of rapidly digestible starch and increased the amount of resistant starch in extruded starches. 

The development of edible and biodegradable packaging technologies is supported by pro-environmental trends, the availability of natural raw materials, and their relatively low cost [22], as well as functional properties that enhance their biocompatibility in food systems [23,24]. Consequently, such materials constitute a viable alternative to conventional packaging made from non-biodegradable materials. Packaging based on natural raw materials may find applications not only in the food industry but also in the cosmetic and pharmaceutical sectors [22,25,26].

It is well-known that classic film-forming solutions typically consist of a matrix and a plasticizer [27,28]. The matrix, in turn, is composed of structural components, the key among which are biopolymers, including starch and its modifications that we have studied. Another main goal of the research described in this paper was the physicochemical characterization, with particular emphasis on the surface properties, of films obtained from potato starch extrudates before and after modification with two types of oils. For the first time, edible vegetable oils, rapeseed or sunflower, were used as plasticizers at various concentrations. Similar studies have shown that the lubricating effect of added plasticizer (palm oil or coconut oil) can also reduce the expansion degree of extruded starch, which in turn leads to an increase in bulk density, hardness, and the water adsorption index [21]. The permeability and colour changes in extruded starch films were determined, along with their thickness, moisture content, solubility, hardness, and deformation resistance, as a function of oil concentration and type. Additionally, FTIR studies of the films were conducted, comparing them with similar studies of the powders from which the films were made.

In addition, the wetting process on extruded starch films was analyzed using various probe liquids of different chemical natures by measuring the advancing and receding contact angles. Based on these measurements, it was possible to determine the contact angle hysteresis and subsequently calculate the surface free energy using the Chibowski (contact angle hysteresis, CAH) model [29,30,31]. The study assumed that the resulting films should be biocompatible and non-toxic because their main ingredients, starch and vegetable oil, exhibit these characteristics and are potentially edible. The catalyst is also approved for use in food products and is often found in leavening agents or baking powders. Furthermore, the extrusion process employs relatively low temperatures, which should not lead to the formation of hazardous by-products, as these temperatures are typical in food processing (e.g., baking). Extrusion is an effective technique, particularly for natural polymers such as polysaccharides. It can induce desirable physicochemical changes in biopolymers, and the resulting starch extrudates can be sustainable ingredients for both food and non-food applications [32]. We hope that the in-depth characterization of films based on starch extrudates will provide tools for controlling the wettability, biocompatibility, and functionality of potential bioneutral/bioinert films intended for contact with food and, in the next stage, enable the use of the tested films in specific food applications.

## 2. Results and Discussion

### 2.1. Physicochemical Characteristics of Extruded Starch Films

#### 2.1.1. Colour and Transparency of Extruded Starch Films

It is well-known that starch, as a natural polymer, is an alternative to plastics in food packaging applications due to its widespread availability, low cost, and biodegradability [33]. Additionally, starch films containing edible oils can be used to increase protection against visible (Vis) radiation. As protective barriers for food, such films may help limit losses and transformations of nutrients, vitamins, and co-pigments. On the other hand, excessively dark films may, in some cases, negatively affect the perception and natural appearance of the protected products. Therefore, knowledge of colour/transparency parameter and its monitoring may prove crucial in specific applications of starch films modified with edible oils. Table 1 presents the results of the colour analysis of starch-based films determined in the CIE Lab* colour space. The analysis included the components L* (lightness), a* (red–green coordinate), and b* (yellow–blue coordinate). Additionally, based on the obtained results, the total colour difference (Δ*E*) relative to the reference samples (C) was calculated.

Sample C exhibited the highest lightness among all analyzed films (L* = 83.96), while simultaneously showing the lowest values of a* (1.34) and b* (5.56), indicating a very light and weakly saturated film.

During the rapeseed oil enrichment process, the Δ*E* values relative to the reference sample were the highest and ranged from ca. 41 to 50, indicating a substantial colour change. For the analogous sunflower oil process, the colour change Δ*E* was clearly smaller, in the range ~ 41–44. The greatest colour difference (Δ*E*) was observed for sample R9 (49.87), whereas the smallest was for S3 (40.50).

The L* parameter, corresponding to lightness, ranged from 44.07 to 55.64. Sample S6 and R6 were the brightest, while R9 was the darkest. These analyses confirm the optical microscopy images of starch extrudates used in the film production process discussed in our earlier paper [19].

Additionally, with increasing oil content in the extrudates, regardless of oil type, an increase in the a* and b* parameters was observed, indicating a gradual increase in the saturation of red and yellow hues. The addition of rapeseed oil during extrusion resulted in films that were distinctly darker compared to those containing sunflower oil. This is particularly evident when comparing values for R- and S-type films with the same oil concentration. For example, the a* value is 10.51 for R3 and 8.73 for S3, whilst the b* value is 31.16 and 25.90, respectively. Analyzing both parameters, it can be concluded that the colour saturation is higher for the samples modified with rapeseed oil: the only deviation from this tendency is a slightly higher value a* obtained for the S9 film (12.01), but it is within the limits of statistical error and comparable to the value for the R9 sample (11.51). Basiak et al. [17] stated that the incorporation of lipids (rapeseed or sunflower oil) into starch extrudates and subsequent film formation results in a more opalescent and glossier appearance compared to unmodified starch films. These observations seem consistent with our research.

From a practical perspective, knowledge of the direction of colour changes in starch films containing edible oils may be utilized to improve protection against visible (Vis) radiation. As protective barriers for food, such films may help limit losses and transformations of nutrients, vitamins, and co-pigments. However, excessively dark films may, in some cases, negatively affect the perception and natural appearance of the protected products.

The transparency of films produced from potato starch extrudates enriched with edible oils was investigated within the visible light range. It is not possible to unequivocally determine an optimal value of transmittance (T), as this parameter may fulfil several key functions in the tested films and is highly dependent on specific applications. Transmittance values approaching 100% indicate nearly complete transmission of radiation in the visible (Vis) spectrum, comparable to the transparency of water. However, achieving such high values is not desirable, as it would result in excessive exposure of packaged products to radiation. This, in turn, may lead to the degradation or oxidation of sensitive compounds such as vitamins, lipids, and pigments, or to protein denaturation. Conversely, complete limitation of visible light transmission (T ≈ 0%) would cause masking of the characteristic colour of food products. Colour is an important attribute influencing consumer purchasing decisions, and its obscuration could negatively affect product acceptance.

**Figure 1:**
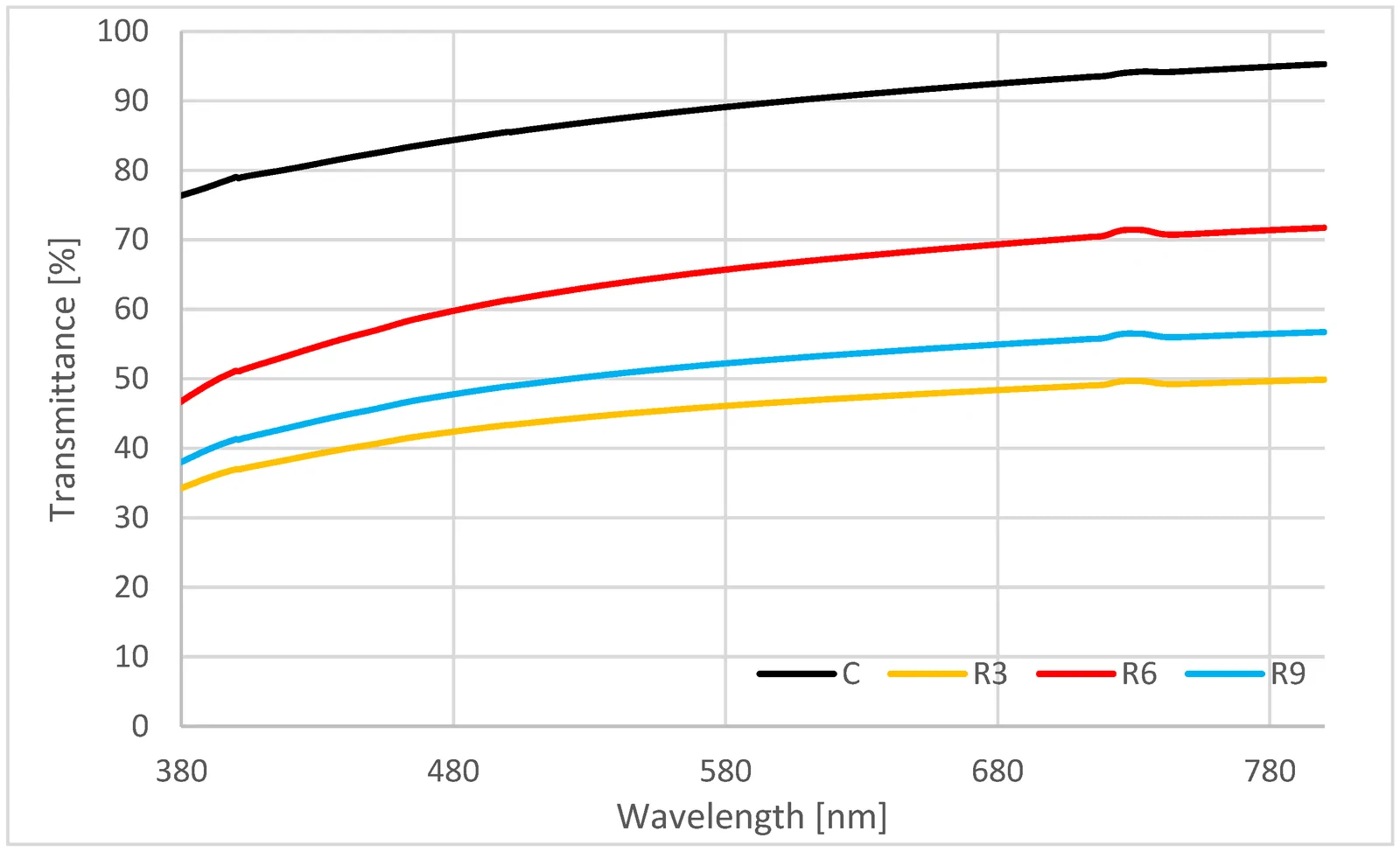
Transmittance of visible light (T) of extruded starch films with and without (C) increasing oil concentrations (R: rapeseed oil, 3% R3, 6% R6, and 9% R9).

**Figure 2:**
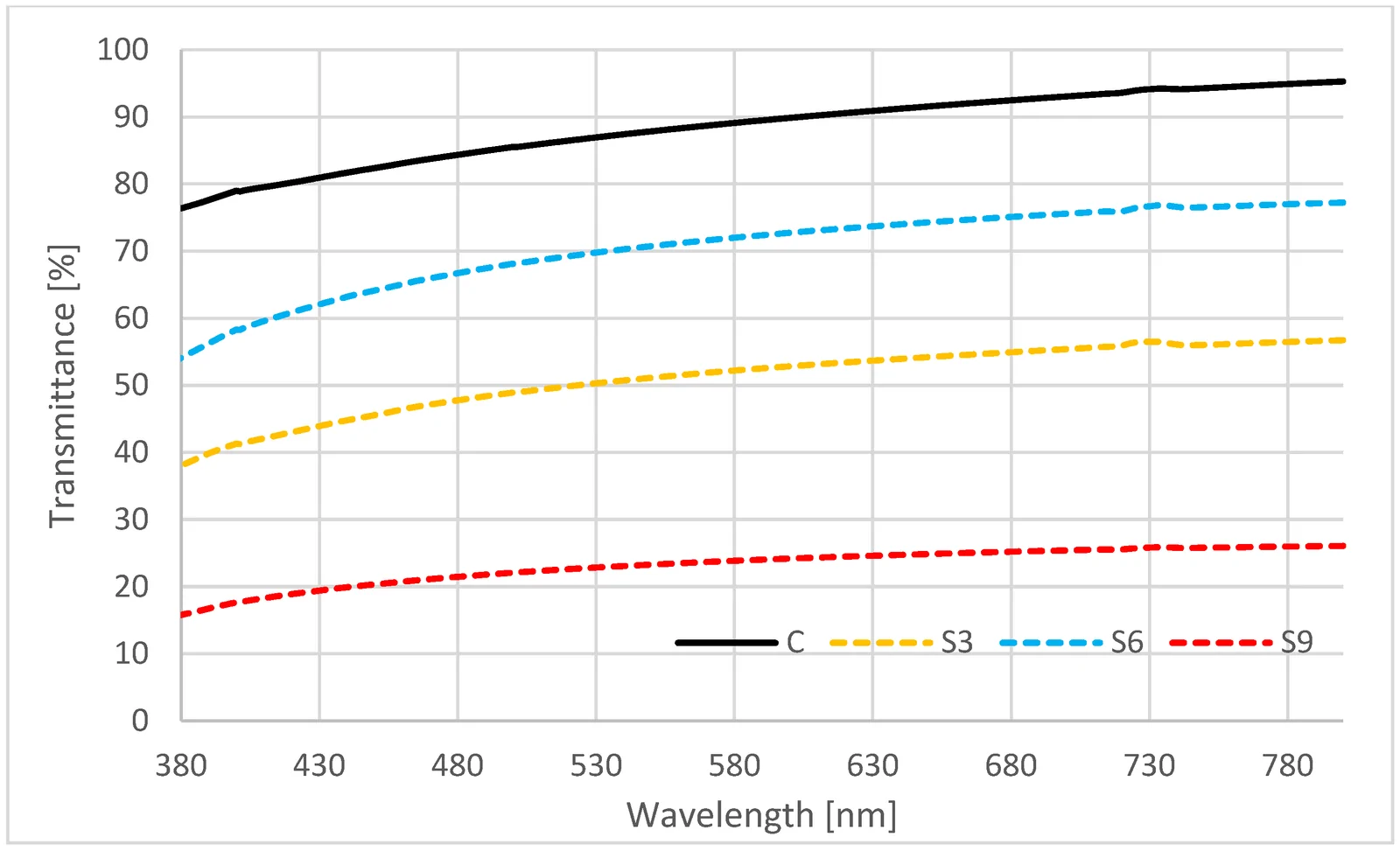
Transmittance of visible light (T) of extruded starch films with and without (C) increasing oil concentrations (S: sunflower oil, 3% S3, 6% S6, and 9% S9).

The film obtained from starch without the addition of edible oil (C) exhibited relatively high transparency in the visible (Vis) radiation range, with transmittance values of T = 76–95%. For modified films, regardless of the type and amount of oil added, the transmittance values were significantly lower, ranging from T = 15% to 77%. The film with the lowest permeability to Vis radiation (T = 15–26%) was identified as sample S9. The intermediate values obtained for R6 and S6 films, averaging around 70% and 77%, respectively, seem to be optimal for our applications (Figure 1 and Figure 2).

The decrease in transparency of the obtained films can be explained by the addition of edible oils, which act as plasticizers in the system [34]. According to Fakhouri [35], the opacity of the films can also be attributed to varying amylose content. Its linear structure mainly creates tight hydrogen bonds between the hydroxyl groups of adjacent chains, which in turn may contribute to reduced biopolymer–water interactions and the formation of an opaque polymer matrix. The limited accessibility of the formed film areas to the water molecules corresponds well to, and explains the decrease in, the solubility of the tested films (Section 2.1.2, Table 2).

Based on the literature review [21,36,37], it can be assumed that the low transmittance values obtained are also related to the formation of amylose–lipid complexes. Amylose incorporates hydrophobic lipid chains into its helical structure. These lipids may carry either positive or negative charges. Their mutual interactions cause starch chains to approach each other closely enough to form hydrogen bonds. Based on the described mechanism of amylose–lipid complex formation, it can be assumed that the type and structure of fatty acids found in rapeseed and sunflower oils also play an important role in the newly formed structures. The function of edible oils as plasticizers is attributed to the so-called “lubrication” effect, which contributes to the increased mobility of amylose chains and promotes double-helix formation, thus leading to the development of semi-crystalline regions [21]. The degree of saturation of the fatty acids likely facilitates the formation of a highly stable, heat-resistant, V-type crystalline conformation [36,37] in both the R and S series. The differences in transmittance result from the number of complexes formed, which in turn is closely related to the bond saturation. Sunflower oil (series S films) is rich in diunsaturated linoleic acid. However, rapeseed oil (series R films) is rich in linear monounsaturated oleic chains [38]. It is also likely that the relatively smaller proportion of edible oil in the film compared to the starch matrix results in the formation of more ordered and higher-density areas, as well as semi-crystalline areas. As a result, a structure is created that acts as a barrier to radiation, leading to a decrease in transmittance (%T) [39]. The formation of amylose–lipid complexes in edible starch films may also have another typical health aspect, i.e., it may decrease the digestibility of starch as a new source of resistant starch (type 5) [40,41].

#### 2.1.2. Thickness, Moisture, and Solubility of Extruded Starch Films

Thickness measurements of films obtained from potato starch extrudates with the addition of edible oils (sunflower or rapeseed oil) revealed noticeable variability (Table 2). The thinnest films were those produced from extrudates without oil addition (C), with a thickness of 0.133 mm. Furthermore, it was demonstrated that extrudates containing 6% oil yielded the thickest films, measuring 0.267 mm (R6, S6).

The highest moisture content was observed in the control sample (C; 13.355). For films containing sunflower oil, an increase in oil concentration resulted in a decrease in moisture content, (S3 > S6 > S9). A comparable level of moisture content was observed among all rapeseed oil-enriched film samples, averaging approximately 11% (Table 2). These subtle changes are also visible in profilometric images, which are closely correlated with a very sensitive parameter, i.e., contact angles discussed in the following Section 2.3.

The solubility dependence of starch extrudate-based films is directly related to the type of oil used to modify them. All oil-enriched films understandably exhibit lower solubility than the reference sample. Films enriched with rapeseed oil are usually characterized by higher solubility compared to those with sunflower oil. Surprisingly, solubility increases with increasing rapeseed oil content, ranging from 3% to 9%. In the case of starch extrudate films enriched with sunflower oil, solubility decreases with increasing concentration. This behaviour can be explained by the different fatty acid profiles of these two vegetable oils [38]. The R-series films exhibit a steady increase in the density of exposed ester groups as a function of concentration [6]. Given their chemical nature, these ester groups are polar, which provide better affinity for and solubility in polar solvents. On the other hand, the S-series films reached supersaturation at 9% loading, likely disrupting optimal structural organization and uniform distribution of surface functional groups. This different oil structural model may result in the different solubility trend. According to Lacerda et al. [22], the introduction of oil can improve the functional properties of the material, e.g., by increasing its hydrophobicity and providing potential antimicrobial activity, thereby expanding its packaging applications. The hydrophobic/hydrophilic nature of the studied starch extrudate systems is discussed in detail in the following Section 2.4.

#### 2.1.3. Hardness and Deformation of Extruded Starch Films

Achieving optimal hardness while maintaining resistance to deformation is an important aspect of film production. These properties enable the prediction of film durability and their potential applicability at various stages of the food supply chain. Obtaining excessively hard films results in low flexibility, which may lead to increased brittleness and reduced resistance to mechanical damage, including deformation.

A classical formulation of film-forming solutions consists of a matrix and a plasticizer; their appropriate qualitative and quantitative selection enables the production of films with desirable adhesion, flexibility, and durability [42,43,44]. The most commonly used structure-forming components are biopolymers such as starch [45]. However, such coatings are usually brittle and prone to cracking, which necessitates the use of plasticizers [45]. Two of the most widely used plasticizers are water and glycerol [46].

In the present study, edible oils (rapeseed and sunflower) were innovatively applied as plasticizers. The addition of rapeseed oil resulted in a significant reduction in film hardness compared to films without oil (C), even by more than two-fold. A similar effect was observed only for the S9 film enriched with sunflower oil. On the other hand, hardness must be discussed in terms of deformation, and here, extruded starch film S6, despite its lower hardness than C (122.00) but still relatively high value (114.00), shows the lowest deformation value of 0.55, i.e., in a similar range to the R-series films of 0.5–0.6. The ratio of relatively high hardness to minimum deformation obtained for sample S6 seems to be optimal among the films tested in terms of potential applications (Table 3).

#### 2.1.4. FTIR Analysis of Extruded Starch Films

**Figure 3:**
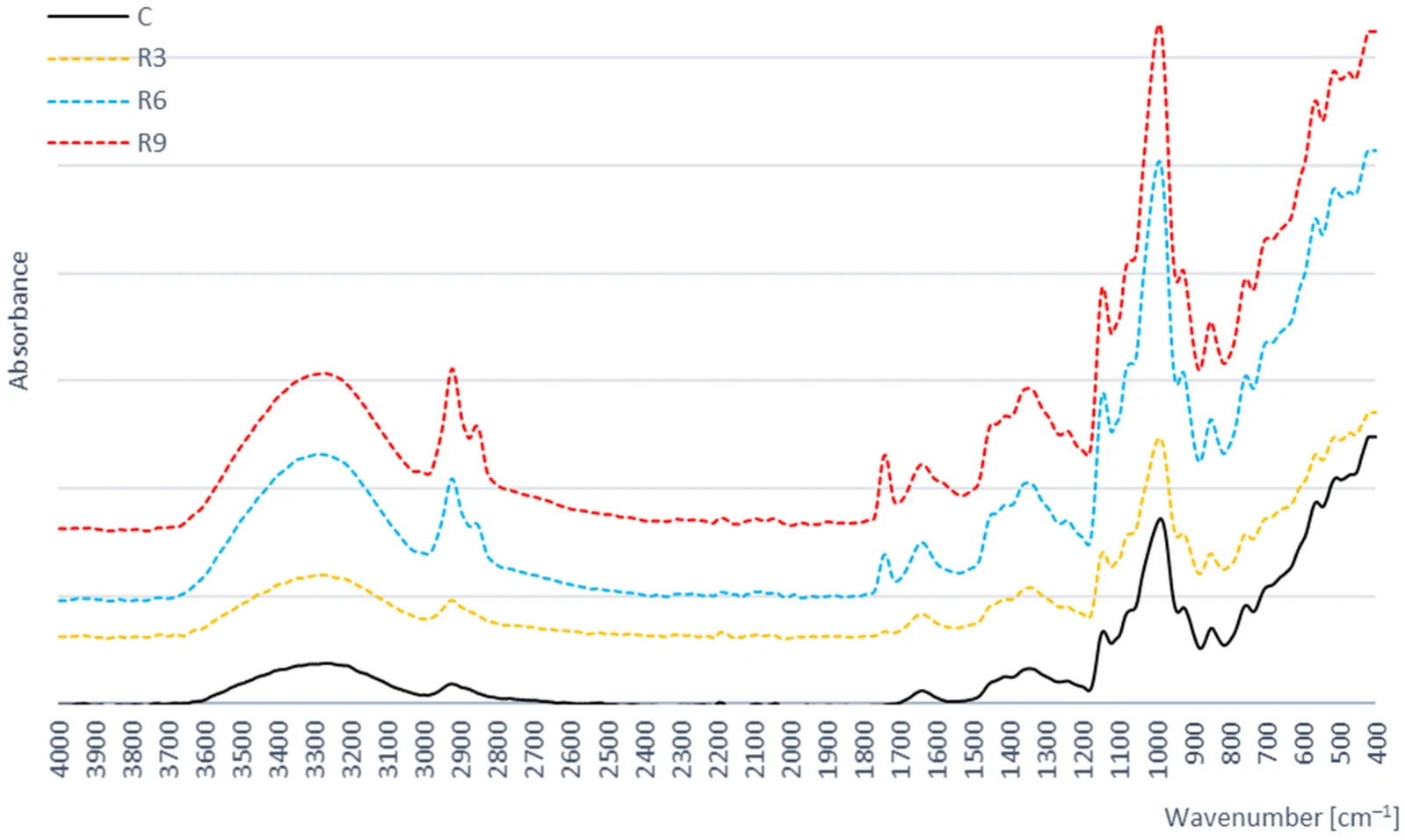
FTIR spectrum of extruded starch films with and without (C) increasing rapeseed oil concentrations (3% R3, 6% R6, 9% R9).

**Figure 4:**
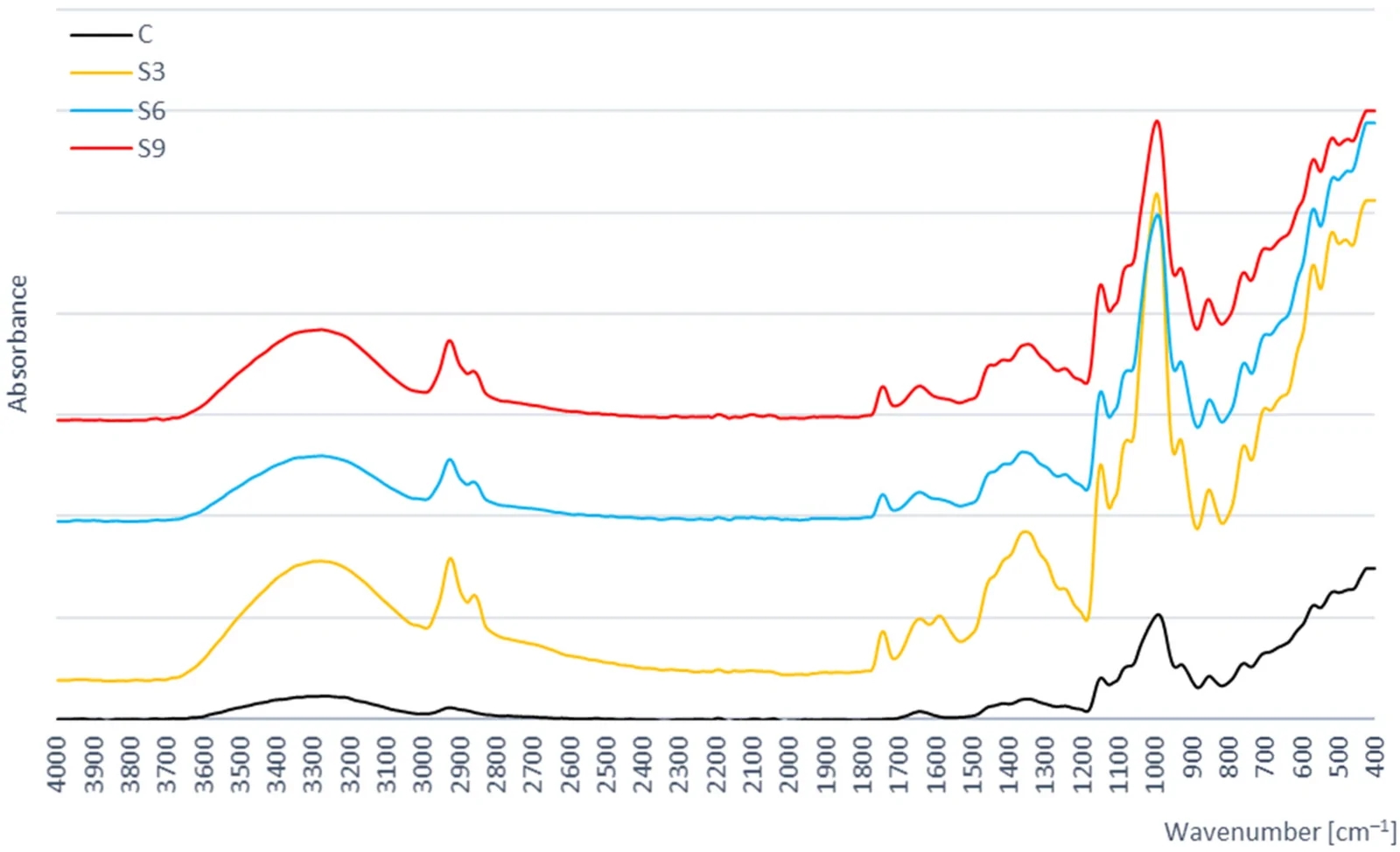
FTIR spectrum of extruded starch films with and without (C) increasing sunflower oil concentrations (3% S3, 6% S6, 9% S9).

The polysaccharide nature of the films is most often confirmed using FTIR spectra [22]. Extruded starch films with and without edible oil were analyzed by FTIR in the range of 400–4000 cm^−1^. Figure 3 and Figure 4 present the obtained measurement results in the form of absorption spectra for samples containing rapeseed oil (R) or sunflower oil (S). These spectra enable the determination of the direction of structural changes resulting from re- and dehydration processes occurring during film preparation.

The obtained absorption spectra were characterized by bands typical for starch–edible oil systems in the ranges of 1170–1250 cm^−1^, 1450–1470 cm^−1^, 1735–1750 cm^−1^, and 2850–2950 cm^−1^, which resulted from the appearance of new peaks or changes in absorbance. Films obtained from extruded starch dispersion with edible oil exhibited a distinct peak at 1742 cm^−1^, which was not observed in the reference samples (C). The presence of this peak indicates the occurrence of the ester carbonyl stretching group C=O [47,48]. Analysis of an analogous extruded starch with and without edible oil, but in powder form instead of the current form of films, also indicated the presence of these characteristic groups, although over a broader range (1741–1743 cm^−1^), as reported in our previous paper [19]. In relation to the above-mentioned results of extrudates in powder form, it was noted that due to their rehydration and re-dehydration during film formation, a decrease in the intensity factor by almost half was observed, in the range 1741–1743 cm^−1^. The intensity coefficient of extruded starch with sunflower oil films ranged from 0.011 to 0.023 (Figure 4), whereas for films with rapeseed oil it ranged from 0.012 to 0.020 (Figure 3), suggesting comparable complexation of the oils. Moreover, the disappearance of the peak at 1742 cm^−1^ was observed for sample R3 compared to the spectrum of the extrudate presented in powder form in the previous study [19]. A possible reason for the disappearance of the band corresponding to the presence of carbonyl groups (C=O), characteristic of esters, may be the hydrolysis of esters or their degradation during thermal processing (including extrusion). The low content of external (0.006 g/100 g) and internal (0.713 g/100 g) lipids in our study during the determination of the degree of complexation [20], as well as subsequent processes such as the grinding of extrudates and film preparation, may explain the disappearance of the aforementioned band.

Another characteristic region indicating the presence of CH_2_/CH_3_ groups, associated with deformation of aliphatic chains in the ester structure, is the peak in the range of 1450–1470 cm^−1^ [47,49]. Additionally, a broad band with two peaks at 2850–2950 cm^−1^ was observed for extruded starch films with and without edible oil (Figure 3 and Figure 4), resulting from the presence of C–H stretching vibrations and bands typical for –CH_2_– and –CH_3_ groups in aliphatic chains. This observation is reflected in the intensity of the peaks often associated with fatty acid esters for the films, for which the increase in oil percentage correlates with absorbance values: (R3) < (R6 and S6) ≤ (R9 and S9).

The appearance of a new band at 1642 cm^−1^ was also observed, which had not been detected for extruded starch in powder form described in the previous study [19]. The band in the range of 1630–1650 cm^−1^ is attributed to the presence of water in the sample, which is expected in the case of the analyzed films. The step-by-step procedure for producing films from aqueous starch extrudate dispersions, described in the experimental part, justifies the appearance of this peak. Additionally, it is well-known that the ratio of absorption intensities at 1047 cm^−1^/1022 cm^−1^ can be used as an indicator of changes in starch crystallinity regions [49]. Similar studies conducted by the Raphaelides research group confirm that the degree of crystallinity of native starch extrudates was lower than that of their lipid-containing counterparts [27].

The highest value of this ratio was recorded for sample S3 (intensity ratio 1047/1022 = 1.0241) (Table 4), which may suggest the formation of a new, highly ordered structure as a result of re- and dehydration during film preparation. For the S6 and S9 films and for all R-series films, similar values of this ratio were obtained in the range of approx. 0.70–0.75, which is indirectly confirmed by earlier studies on the same extrudates but in powder form, with values of 0.70–0.75 [19].

**Figure 5:**
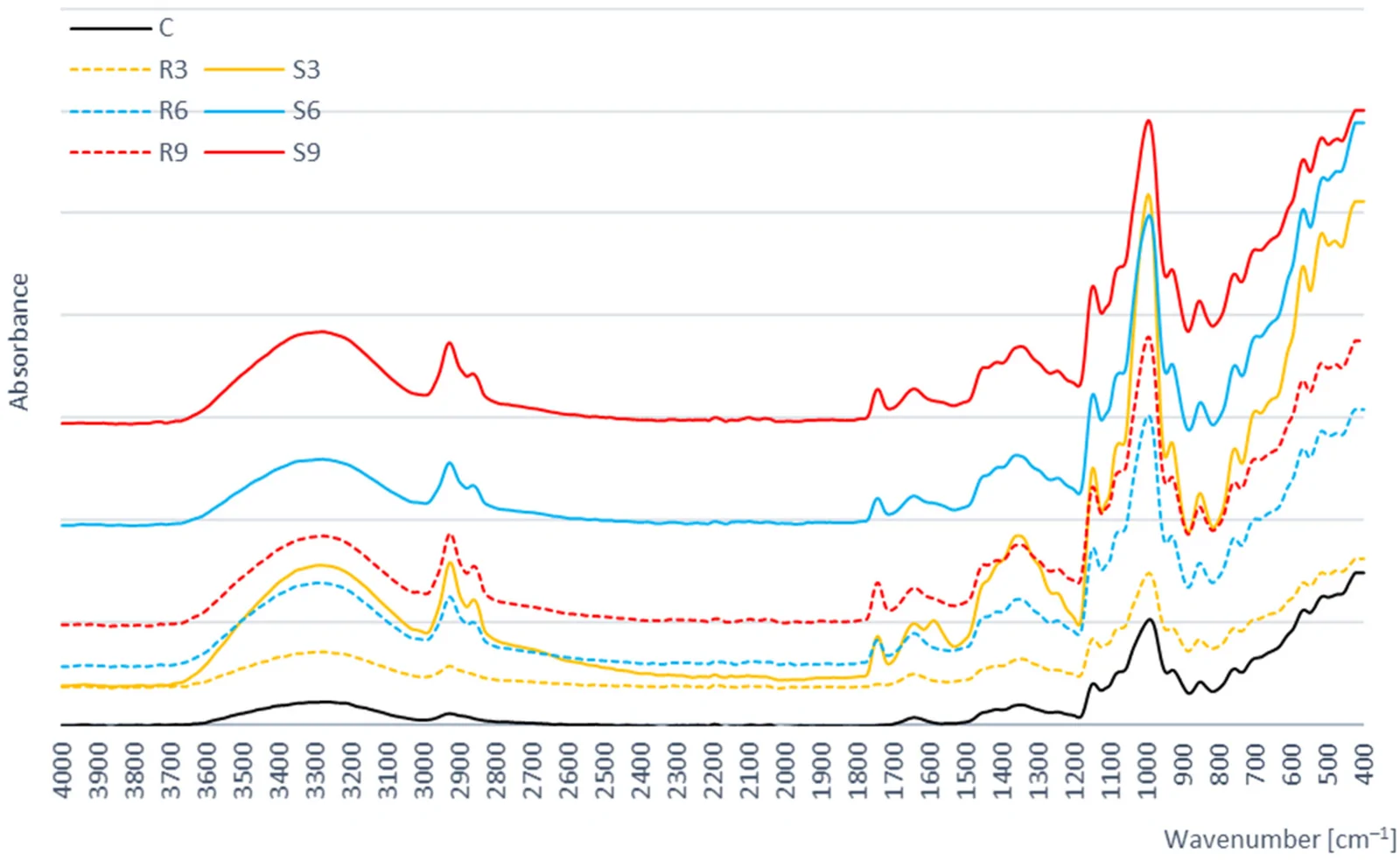
FTIR spectrum of extruded starch films with and without (C) increasing oil concentrations (R: rapeseed oil, S: sunflower oil, 3% R3 or S3, 6% R6 or S6, 9% R9 or S9).

For better visualization, a figure summarizing both series of extrudates modified with rapeseed and sunflower oil is provided below (Figure 5).

### 2.2. Profilometric Analysis of Extruded Starch Films

**Figure 6:**
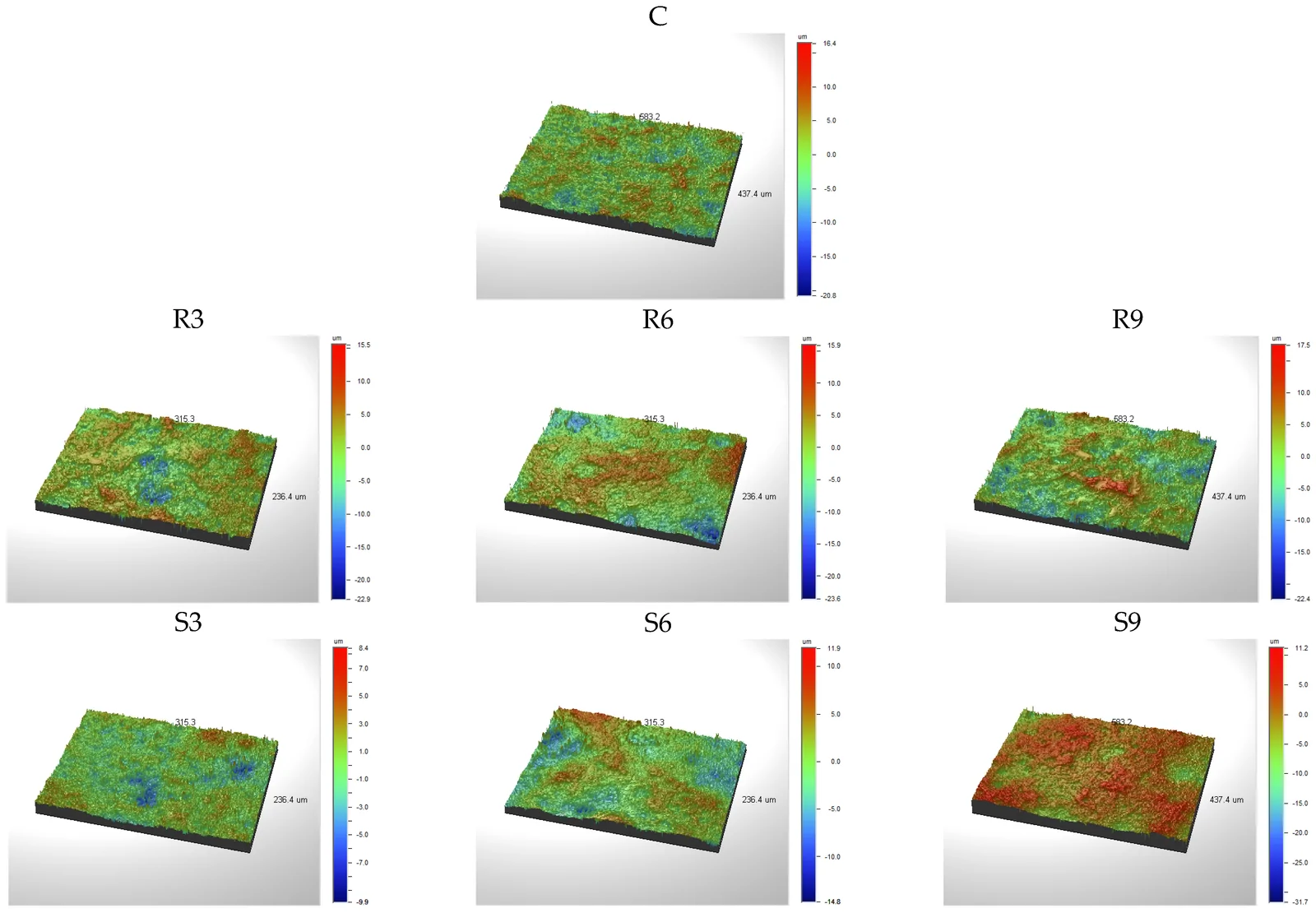
Profilometric images depicting the surface topography of extruded starch films with and without (C) increasing oil concentrations (R: rapeseed oil, S: sunflower oil, 3% R3 or S3, 6% R6 or S6, and 9% R9 or S9).

The optical profilometer used enables rapid, non-contact, three-dimensional surface magnification. It can be used to measure materials with both very high and very low roughness. The extruded starch film images from the optical profilometer are presented in Figure 6. They show topographical differences between extruded starch film surfaces with and without edible oil. The homogeneity of extruded starch films is strictly oil-concentration-dependent, similar to other parameters (thickness, moisture, solubility, hardness, and deformation) described in Section 2.1.2 and Section 2.1.3. The exposition of bare starch surface or extruded starch with edible oil should contribute to the values of contact angles and surface free energy calculated from them, described in detail in the following parts of the manuscript.

Topographical parameters characterizing the surface of extruded starch films, with and without increasing oil concentration (rapeseed or sunflower), are presented in Table 5. With increasing edible oil concentration, the homogeneity of the extruded starch film decreases because the degree of starch–oil binding increases. This conclusion can be drawn from the increase in the values of all roughness parameters ( $R_{a},$ $R_{q}$ , and $R_{t}$ ) compared to the values for the surface of the base extruded starch film (see C, Table 5). These considerations are consistent with the study by Nisitthichai et al. They found that starch extrudates exhibited poorer expansion and texture with increasing palm oil addition, which was attributed to the lubricating effect of the oil [21].

An increase in the rapeseed oil concentration from 3% to 9% in the case of extruded starch films caused an increase in the topographic values of $R_{a},R_{q}$ , and $R_{t}$ from 2.88 nm to 3.41 nm, from 3.62 nm to 4.26 nm, and from 32.31 nm to 41.64 nm, respectively. In the case of films enriched with sunflower oil, the increase in all roughness parameters was even greater, almost twofold but only for series S3 and S6: from 1.33 to 2.41 for $R_{a}$ , from 1.72 to 3.07 for $R_{q}$ , and from 19.13 to 36.26 for $R_{t}$ . In the case of the S9 film, a slight decrease in the values of $R_{a}\;and\;R_{q}$ was again observed while $R_{t}$ remained at a similar level to S6 (Table 5). This can be explained by the saturation of the sample with oil, which is confirmed by other methods in the studies described in our earlier paper [19].

Consequently, these films are permeable to the tested liquids used to measure contact angles to varying degrees. Based on the obtained contact angle and topographic results, it is possible to infer the extent to which the surface free energy of the studied films is determined by their surface roughness, which will be discussed in detail in Section 2.3 and Section 2.4. Other factors also influence surface parameters, such as the presence of unsaturated bonds in the molecules and the phase state in which these films occur. Therefore, the FTIR spectra of both extruded starch with and without edible oil in the form of powder and the tested films made from their dispersion can be useful, as illustrated in Section 2.1.4. In the next stage (Section 2.3), correlations were sought between changes in surface roughness and the contact angle values measured on the surface of extruded starch films before and after oil enrichment, as well as their surface free energy (Section 2.4).

The results obtained from advanced analytical techniques indicate that by controlling extrusion-processing conditions and adding various edible oils in varying proportions, a range of products with tailored functional properties can be produced. These systems are suitable for potential use as coatings in food and pharmaceutical systems, including edible films and biodegradable materials.

### 2.3. Wettability of Extruded Starch Films

**Figure 7:**
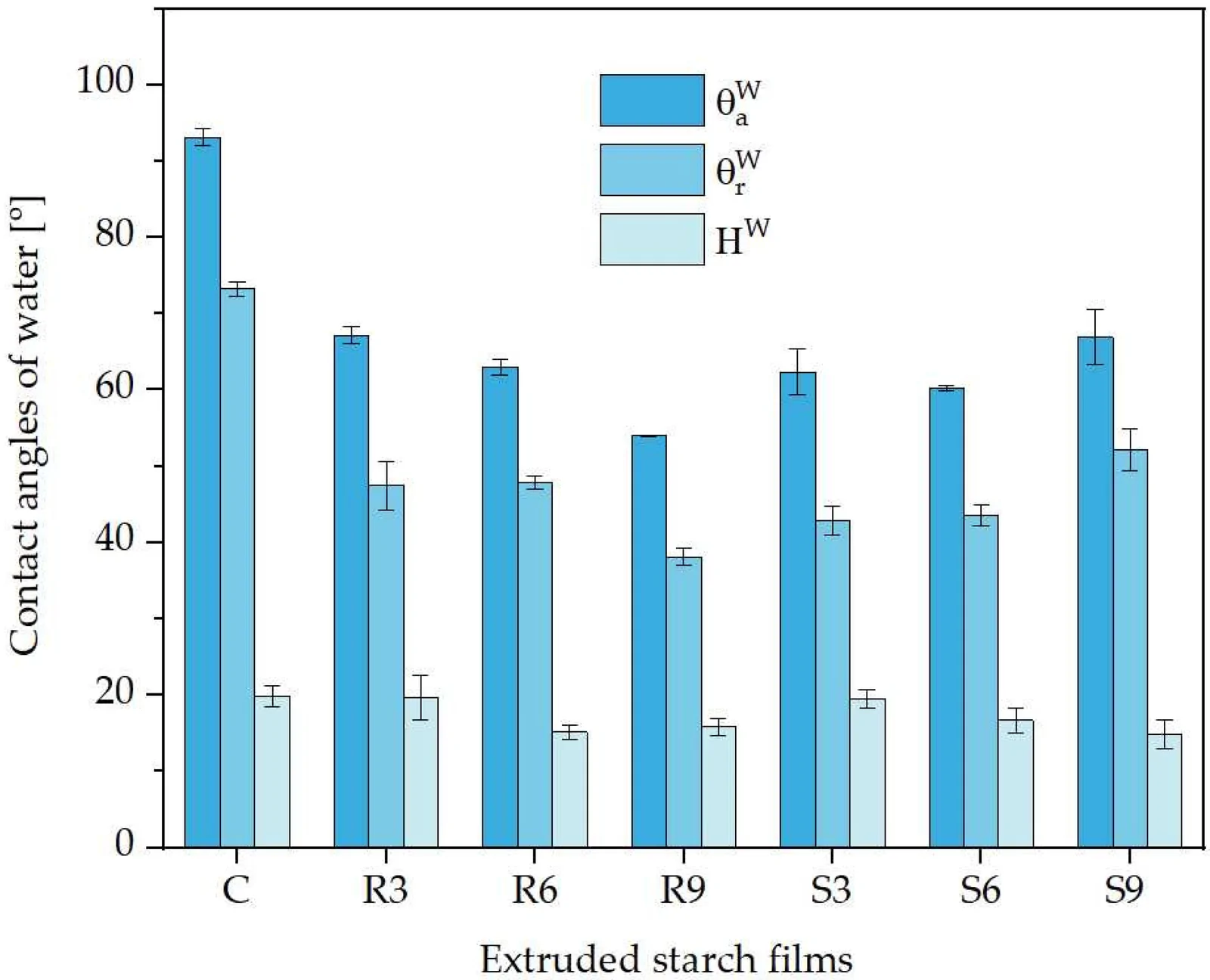
Advancing ( $\theta_{a}$ ) and receding ( $\theta_{r}$ ) contact angles of water (W) and their hysteresis ( $H^{W}$ ) measured on extruded starch films with and without (C) increasing oil concentrations (R: rapeseed oil, S: sunflower oil, 3% R3 or S3, 6% R6 or S6, and 9% R9 or S9).

Contact angle measurements of different liquids can provide a preliminary evaluation of the kind and strength of the interactions across the solid/liquid interface. Water, formamide, and diiodomethane serve as a fundamental triad of probe liquids, each selected for its distinct molecular interaction profile during advancing and receding contact angle measurements [31,50]. Water acts as the primary polar reference. When measuring the advancing contact angle, water reveals the initial wettability, while the receding contact angle often highlights the material’s high affinity for moisture and its tendency to swell, typically resulting in significant contact angle hysteresis defined as the difference between the advancing and receding contact angle. The advancing and receding contact angles of water and their hysteresis measured on starch extrudate-based films are listed in Figure 7.

The modification of starch extrudates with edible oils significantly altered their wetting behaviour. On the one hand, it is surprising that the incorporation of the oil phase reduced the advancing contact angle from ca. 93° for the reference sample (C) to 54–67° for R samples and 60–67° for S samples, indicating enhanced surface hydrophilicity. On the other hand, it may mean that oil accumulates inside the starch grains or in the free spaces, thereby exerting a fluidizing effect that may facilitate a more effective penetration of the liquid into the deeper layers of the film. Consequently, the droplet behaviour is governed by a combined mechanism. In such systems, initial surface wetting is accompanied by simultaneous liquid absorption and capillary-driven migration (wicking) into the bulk matrix. Capturing this dual behaviour provides a far more comprehensive characterization of the modified extrudates, demonstrating that the measured contact angles are highly sensitive to chemical composition and structural architecture across the surface and the film interior. The obtained values, while relative and apparent, remain comparable and methodologically reliable for evaluating the wettability of the studied systems.

This interpretation is consistent with the contact angle hysteresis ( H ) trends. A significant decrease in H values was observed, from 19.9° for the reference sample (C) to approximately 15° at 6% and 9% oil content (R6 and R9). Similarly, H values for the S series gradually decline to around 15° (S9). In contrast, a low oil concentration (3%) yielded no significant changes for R3 (19.7°) or S3 (19.5°). For both the native starch extrudate (C) and the 3% oil samples, the higher hysteresis was primarily driven by rapid swelling and surface hydration upon contact with water. Incorporating higher amounts of edible oil (6% and 9%) partially mitigated this hygroscopic behaviour, thereby stabilizing the three-phase contact line which is crucial during wettability measurements.

As mentioned previously, the effect of edible oils is primarily attributed to their function as plasticizers via the so-called “lubrication” effect. By increasing the mobility of amylose chains, the oil facilitates double-helix formation and structural reorganization leads to the formation of regions with distinct local polarity, driven by the differential exposure of either hydrophilic functional groups of starch or hydrophobic fatty acid chains. This arrangement directly governs the balance between liquid spreading and wicking, thereby dictating the overall wetting properties of the modified films [21].

Optical profilometry also revealed distinct structural differences for extruded starch films as a function of edible oil type on a nanometre scale. A concentration-dependent increase in the roughness parameters ( $R_{a}$ , $R_{q}$ , $R_{t}$ ) of films was observed for the rapeseed oil (R) series. Conversely, modification with sunflower oil generated a dual behaviour: a decrease in film roughness at 3% concentration (S3), followed by a subsequent increase at higher concentrations (S6 and S9). These divergent trends in surface roughness and wetting behaviour are directly linked to the distinct fatty acid profiles of the two edible oils [38], which dictate the spatial packing and conformation of the grafted ester chains. Importantly, the fact that hysteresis decreased for R9 and S9 films despite their increased roughness confirms that the surface energetic state is predominantly governed by chemical changes, likely due to dominance of an oil phase masking the starch matrix, rather than by its topography. Our results correlate well with published data [17], demonstrating that oils decreased moisture absorption, particularly at higher relative humidity, and reduced the surface swelling index when in contact with water droplets. This mechanism effectively contributes to the promising dual hydro/lipophilic character of extruded starch enrichment with edible oil, which we have already indicated in our preliminary studies in a previous paper [19].

**Figure 8:**
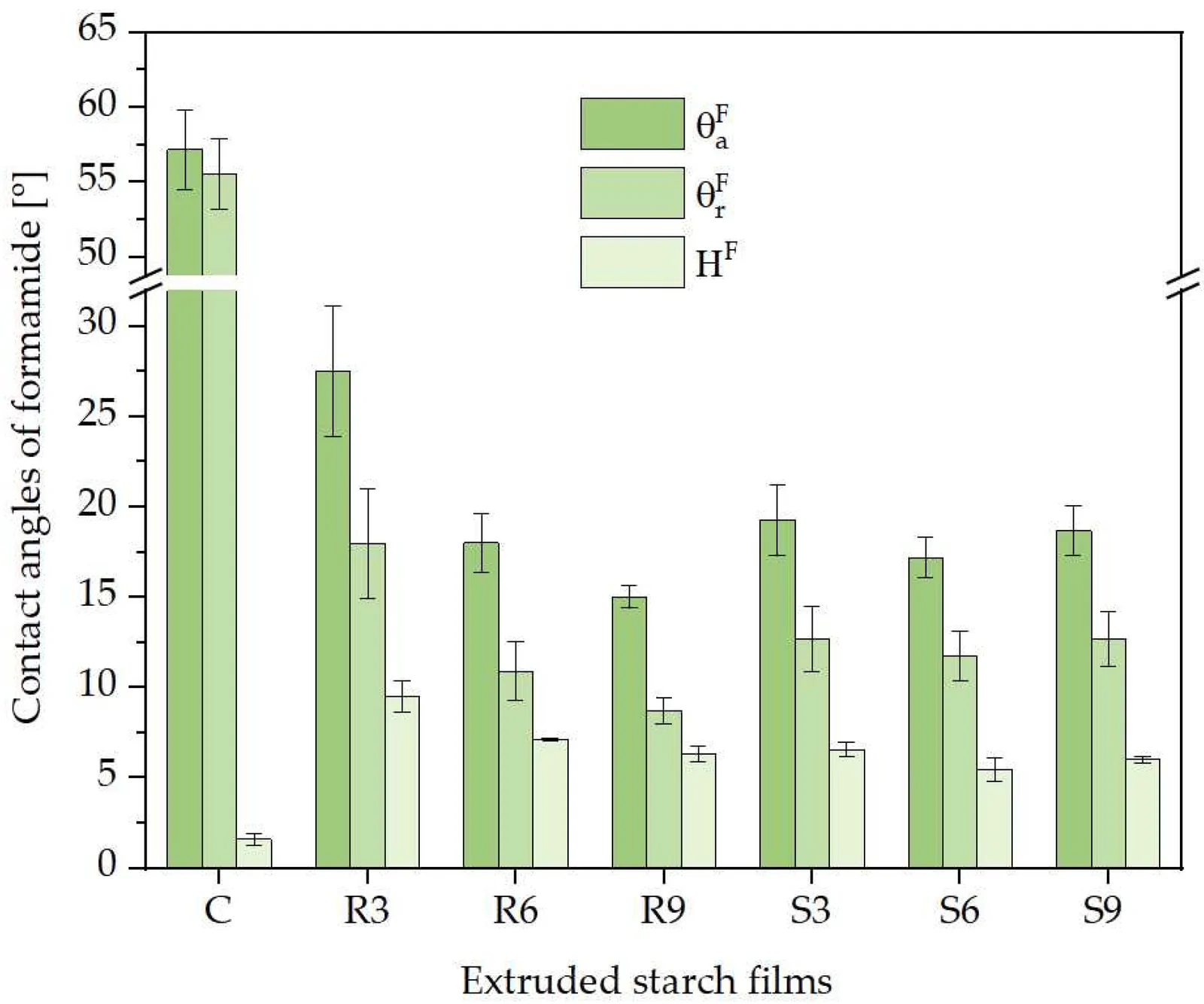
Advancing ( $\theta_{a}$ ) and receding ( $\theta_{r}$ ) contact angles of formamide (F) and their hysteresis ( $H^{F}$ ) measured on extruded starch films with and without (C) increasing oil concentrations (R: rapeseed oil, S: sunflower oil, 3% R3 or S3, 6% R6 or S6, and 9% R9 or S9).

To further elucidate the surface energetic profile and the nature of the oil modification, contact angle measurements were performed using formamide as a complementary polar probe liquid. Formamide possesses a lower surface tension (58.0 mN/m) and different donor–acceptor capabilities compared to water. This allows formamide to penetrate the polymer matrix differently than water, providing a more detailed and extended insight into the acid–base interactions at the interface. Its advancing and receding contact angles are presented in Figure 8.

For the reference sample (C), the advancing contact angle of formamide was 57.2°. Upon modification with vegetable oils, a drastic reduction in advancing contact angles was observed, to ranges of 15.0–27.5° for the rapeseed oil (R) series and 17.2–19.3° for the sunflower oil (S) series, respectively. This is in close agreement with literature reports stating that the addition of rapeseed oil consistently decreases the contact angle for all tested liquids except water. This modification increases the surface affinity of the films toward less polar liquids [17]. Given that formamide possesses a lower surface tension (58.0 mN/m) than water (72.8 mN/m), this wide spreading behaviour, combined with the strong electron-donor character of liquid, indicates that oil-modified extrudate starch surface presents highly accessible sites capable of Lewis acid–base interactions.

Interestingly, the contact angle hysteresis ( H ) of formamide exhibited an upward trend, increasing from a negligible 1.6° for the control sample (C) to 5.5–9.5° for the oil-modified samples. For the control, the minimal hysteresis coupled with a relatively high advancing contact angle demonstrates a homogeneous, rigid, and densely interconnected native starch extrudate matrix that prevents any immediate solvent (probe liquid) penetration or swelling. Conversely, the significant increase in hysteresis upon oil incorporation (R and S series) reflects the enhanced macromolecular flexibility induced by the fluidizing effect of the oil phase. This phenomenon increases the free volume of the esterified starch chains, enabling localized conformational movements or penetration of the probe liquid during wetting. Consequently, during the receding contact angle measurement, the formamide contact line becomes pinned by strong Lewis acid–base interactions within the ester-rich matrix, lowering the receding contact angle and increasing the hysteresis.

Notable distinctions between the two edible oils further support this structural model. The R-series films displayed a continuous, concentration-dependent reduction in advancing contact angles, reaching 15.0° at 9% oil content (R9). This indicates a steady increase in the density of exposed ester groups and/or film fluidity, thereby facilitating liquid spreading across the surface and penetration into the films. Conversely, the S-series films reached their minimum contact angle at 6% (S6), with a slight increase at 9% (S9). This non-linear trend aligns perfectly with the optical profilometry data, suggesting that at 9% sunflower oil loading, an over-saturation state was reached. This over-saturation likely induced micro-phase separation or disrupted the optimal structural organization and uniform exposure of the surface functional groups.

**Figure 9:**
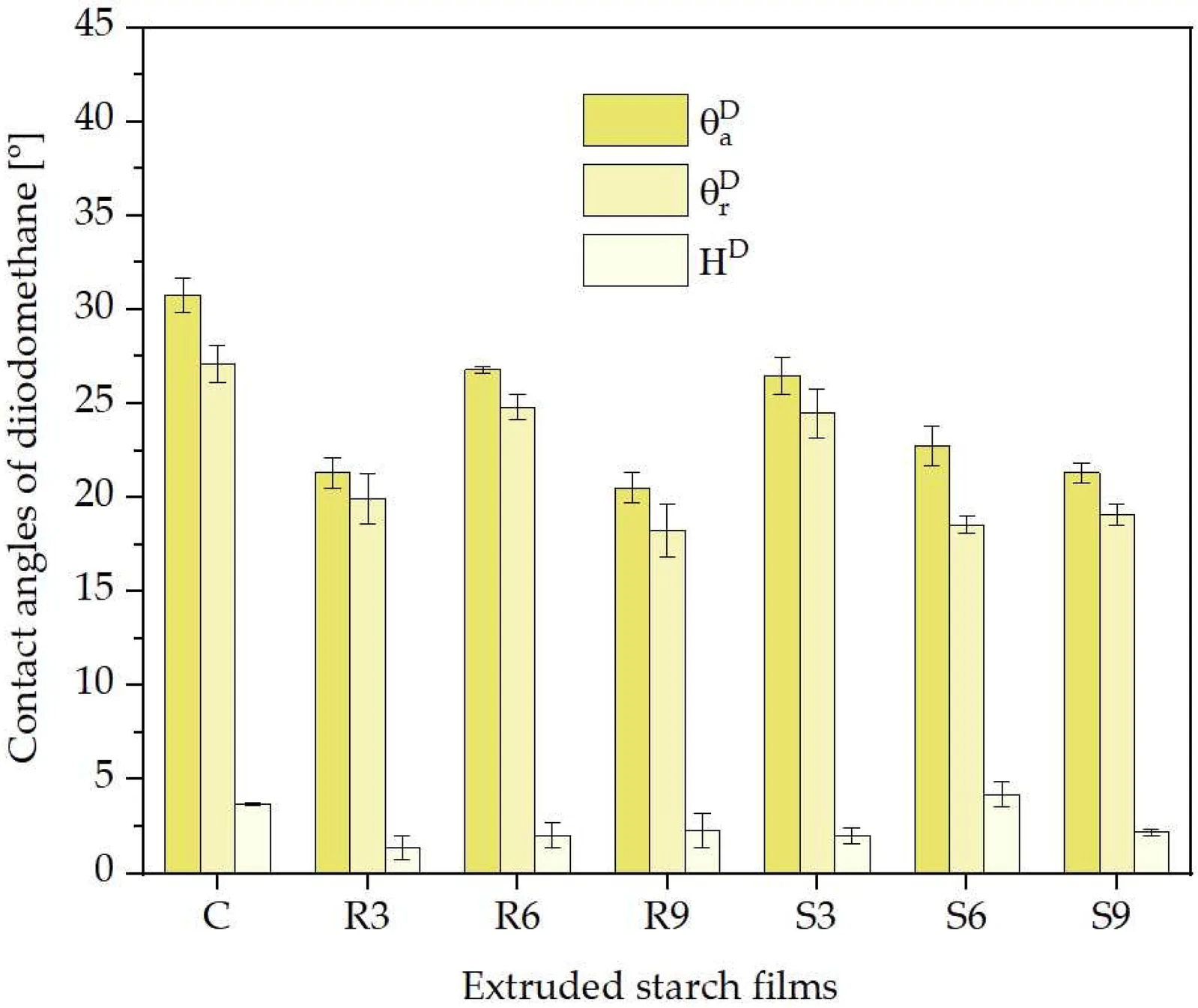
Advancing ( $\theta_{a}$ ) and receding ( $\theta_{r}$ ) contact angles of diiodomethane (D) and their hysteresis ( $H^{D}$ ) measured on extruded starch films with and without (C) increasing oil concentrations (R: rapeseed oil, S: sunflower oil 3% R3 or S3, 6% R6 or S6, and 9% R9 or S9).

Diiodomethane contrasts these two probe liquids as a purely dispersive (non-polar) liquid with a surface tension of 50.8 mN/m. Since it lacks the ability to form hydrogen bonds, its advancing and receding angles are governed almost exclusively by Lifshitz–van der Waals forces. Consequently, its behaviour is relatively unaffected by the density of polar groups, providing a direct assessment of the surface lipophilicity and the density of non-polar functional groups. The measured advancing and receding contact angles of diiodomethane and their hysteresis are listed in Figure 9.

The control starch film (C) displays the highest advancing (30.8°) and receding (27.1°) contact angles, combined with a baseline hysteresis of 3.7°, confirming that the unmodified polysaccharide matrix has the lowest affinity for the non-polar phase. Chemical modification via extrusion with edible oils incorporates long-chain fatty acid residues into the starch backbone through esterification [19]. This drives the noticeable reduction in contact angles across the modified series, confirming a successful transition toward a more lipophilic surface state.

As mentioned previously, the two edible oils alter the surface topography and wetting kinetics through fundamentally different mechanisms, related to their fatty acid profiles [38]. For the rapeseed oil (R) films, characterized by more linear monounsaturated oleic chains, a concentration-dependent increase in nanoscale roughness directly modulates droplet behaviour. At 3% oil incorporation (R3), the homogeneous arrangement of the grafted ester chains causes a sharp drop in contact angles (21.3°) and a minimal hysteresis of 1.4°. At 6% (R6), however, the progressive increase in nanoscale roughness introduces a geometric pinning effect, temporarily raising the contact angles (26.8°) while maintaining a low hysteresis (2.0°). At 9% concentration (R9), the chemical dominance of the dense, substituted ester network combined with the fluidizing free oil drives the contact angles to their lowest values (20.5°).

Conversely, the sunflower oil (S) films, rich in diunsaturated linoleic acid [38], exhibit a non-linear topographic evolution that strongly correlates with changes in hysteresis. At 3% concentration (S3), the oil initially acts as a smoothing agent, reducing the surface roughness below the control baseline (C), which yields a relatively high advancing angle (26.5°) and a steady hysteresis of 2.0°. As the oil content increases to 6% (S6), this trend reverses, and the roughness rises well above the control value. This transition introduces substantial surface heterogeneity, reflected as a sharp increase in hysteresis to 4.2°. At 9% (S9), the system reaches a steady state with a more uniform exposure of the non-polar groups, resulting in a linear decrease in contact angles (21.3°) and a stabilized hysteresis of 2.2°.

In conclusion, both edible vegetable oil modifications successfully enhance the lipophilic character of the starch matrix, which is highly beneficial for improving the moisture barrier properties of biopolymer packaging, but in a slightly different way. Therefore, to better understand the mechanism of these modifications and to gain deeper insights into the nature and magnitude of film surface–liquid interactions, the subsequent section will discuss the alterations in surface free energy, determined based on measured contact angles.

### 2.4. Surface Free Energy of Extruded Starch Films

**Figure 10:**
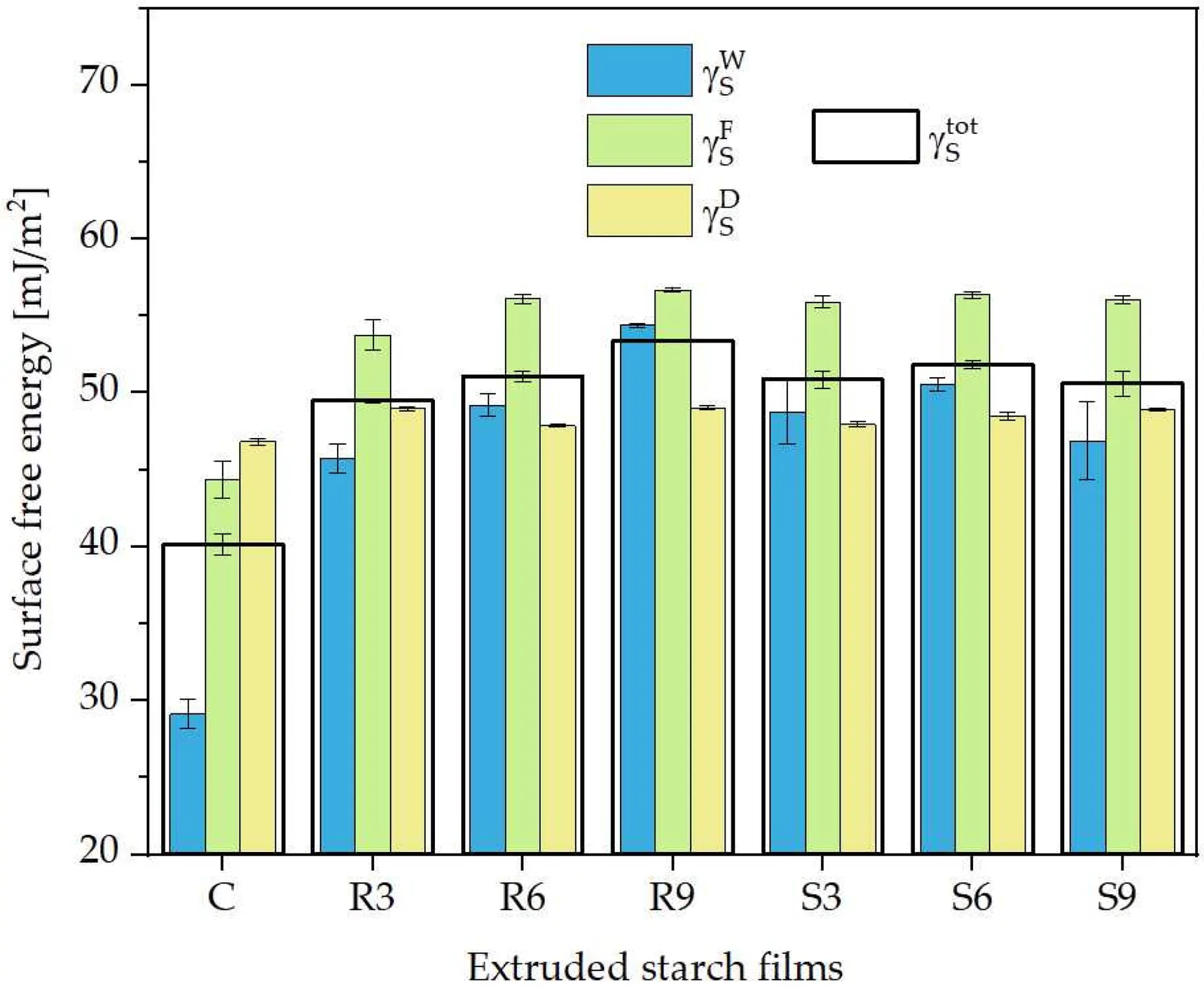
Surface free energy calculated from the contact angle hysteresis (CAH) of water ( $\gamma_{S}^{W}$ ), formamide ( $\gamma_{S}^{F}$ ), and diiodomethane ( $\gamma_{S}^{D}$ ), along with their averaged value ( $\gamma_{S}^{tot}$ ), for extruded starch films with and without (C) increasing oil concentrations (R: rapeseed oil, S: sunflower oil, 3% R3 or S3, 6% R6 or S6, and 9% R9 or S9).

Surface free energy reveals the specific type and strength of interactions occurring between extrudate films and liquids. To determine this interaction strength, the CAH model utilizes only three parameters: the advancing and receding contact angles, combined with the surface tension of a single probe liquid [29,30,31]. In this study, surface free energy was calculated separately from the contact angle hysteresis of water ( $\gamma_{S}^{W}$ ), formamide ( $\gamma_{S}^{F}$ ), and diiodomethane ( $\gamma_{S}^{D}$ ), which were then averaged to determine the total surface free energy ( $\gamma_{S}^{tot}$ ). The obtained values are presented in Figure 10. These results are considered apparent values because their underlying components, including Lifshitz–van der Waals forces, hydrogen bonds, and other interfacial interactions, vary depending on the specific probe liquid used.

Regardless of the probe liquid used, the surface free energy calculated for the oil-free control films (C) was consistently lower than that of the oil-containing films, indicating weaker surface interactions. When evaluated using water contact angle hysteresis, the surface free energy of probe C was determined to be 29.1 mJ/m^2^. The incorporation of edible oils significantly increased these values, reaching 46–54 mJ/m^2^ for films with rapeseed oil (R) and 47–51 mJ/m^2^ for those with sunflower oil (S). Notably, the R9 sample exhibited the highest $\gamma_{S}^{W}$ value, indicating the strongest interaction with water.

This increase in surface free energy and polar affinity upon the addition of hydrophobic oils can be attributed to the specific chemical and structural reorganization of the modified extrudates. Given the high degree of substitution (DS ≈ 2.5) and FTIR data confirming esterification, for starch extrudates in the form of powder [19] and films presented in Section 2.1.4, this process successfully replaces most of the native starch hydroxyl groups with ester linkages. Although the fatty acid aliphatic chains are hydrophobic, the newly introduced carbonyl groups (C=O), originating from both the grafted starch esters and the residual free triglycerides, possess a strong dipole moment, acting as effective hydrogen bond acceptors for water molecules. These interactions can be facilitated by the fluidizing effect of the oil phase, which enhances the mobility of the macromolecular chains. Consequently, the polar ester groups, alongside the remaining unesterified -OH groups, can be highly accessible at the outermost surface layer. Furthermore, this oil-induced fluidization increases the free volume within the polymer network, which may facilitate a more efficient penetration of the probe liquid into the deeper layers of the film during contact angle measurements, thereby affecting the calculated $\gamma_{S}^{W}$ values.

The changes in surface free energy values derived from the different liquids further elucidates the specific nature of these surface interactions. Regarding the $\gamma_{S}^{F}$ values calculated from the formamide contact angle hysteresis, the control sample C yielded 44.4 mJ/m^2^, while the oil-modified films showed very similar values, oscillating around 56 mJ/m^2^. The only exception was the R3 sample, which displayed a lower value of 53.8 mJ/m^2^, indicating weaker interactions of this type. Unlike water, which possesses perfectly balanced electron-donor and electron-acceptor components ( $\gamma_{L}^{+}=\gamma_{L}^{-}$ ), formamide features a highly asymmetric nature dominated by a strong electron-donor ( $\gamma_{L}^{-}$ ) capability. Consequently, formamide interacts significantly more intensely with Lewis acids. In the modified extrudate films, these acidic centres can be primarily represented by the starch hydroxyl groups (-OH). Concurrently, the minor electron-acceptor ( $\gamma_{L}^{+}$ ) component of formamide pairs with the electron-donating carbonyl groups (C=O) of the starch esters and residual triglycerides. This acid–base complementarity drives the significant increase in surface free energy $\gamma_{S}^{F}$ .

Both water and formamide are highly polar liquids capable of participating in strong hydrogen bonding and acid–base interactions. The pronounced changes in surface free energy observed demonstrate that the addition of oil primarily enhances the polar component of the surface free energy, driven by the exposed ester linkages, the polar groups of free lipids, and the oil-induced extrudate film fluidity that eases polar liquid penetration. Furthermore, it should be emphasized that both polar probe liquids also exhibit significant dispersive components—21.8 mN/m for water and 39.0 mN/m for formamide. Consequently, dispersive interactions between these polar liquids and the modified extrudates directly influence the magnitude of the total surface free energy as well. On the film side, these non-polar interactions are driven not only by the newly introduced, hydrophobic aliphatic chains of the fatty acids but also by the hydrocarbon backbone of the starch extrudates. To precisely evaluate the magnitude of these specific non-polar forces, the wetting behaviour and contact angle hysteresis of diiodomethane as a purely dispersive liquid were subsequently analyzed.

The surface free energy values calculated from non-polar diiodomethane contact angle hysteresis ( $\gamma_{S}^{D}$ ) showed essential increases, rising from 46.8 mJ/m^2^ for the control sample C to a narrow range of 47.9–49.0 mJ/m^2^ for the oil-modified films. As mentioned above, extrusion in the presence of edible oils induces the esterification of the starch, successfully grafting long-chain fatty acid residues onto the biopolymer backbone [19]. This substitution alters the surface energetic state. The hydrophobic, aliphatic hydrocarbon tails of the grafted fatty acids are easily accessible for probe liquid, significantly enhancing the dispersive intermolecular interactions with diiodomethane. Consequently, the noticeable increase in surface free energy of the oil-modified series provides clear evidence of an enhanced lipophilic surface character while maintaining the hydrophilic character of the starch matrix.

When considering the total surface free energy $\gamma_{S}^{tot}$ (calculated as the arithmetic mean of the values obtained from water, formamide, and diiodomethane hysteresis), the control sample C showed a value of 40.1 mJ/m^2^. For the rapeseed oil (R) series, a clear concentration-dependent trend is visible, with $\gamma_{S}^{tot}$ systematically rising from 49.5 to 53.4 mJ/m^2^. The R9 sample exhibits the highest value. This suggests that higher concentrations of rapeseed oil can promote more extensive fluidization and structural rearrangement, thereby altering the accessibility of hydrophilic groups for the probe liquids. In contrast, the sunflower oil (S) series shows a plateau effect, with $\gamma_{S}^{tot}$ values stabilizing around 50.6–51.8 mJ/m^2^. The maximum value was achieved for S6, implying that surface saturation occurs at lower inclusion levels, and further increases in oil content do not significantly alter the balance of polar and dispersive interactions at the film surface. 

These values closely align with literature reports, where the surface free energy calculated using the Owens–Wendt–Rabel–Kaelble method ranged from 51.64 to 70.81 mJ/m^2^ for oat starch-based and corn starch-based films [51]. Furthermore, this type of analysis, including polar and dispersive interactions, was successfully employed in another study [8] to evaluate the effect of oil addition on the final properties of potentially edible films based on starch extrudates, aiming to identify directions for improving their properties. This approach provides a valuable framework for our work, as the next stage of our planned research will focus on the optimal application of these films for food coating and/or packaging. The optimization of the surface free energy values effectively establishes a promising dual hydrophilic-lipophilic character in the oil-modified starch extrudates. This can enhance the material compatibility with diverse matrices, making it a highly valuable candidate for advanced functional applications.

## 3. Materials and Methods

### 3.1. Materials

#### 3.1.1. Modification of Extruded Potato Starch with Edible Oil

Extrudates were obtained from potato starch (Potato Industry Plant “Lublin”, Lublin, Poland) and both edible oils: rapeseed or sunflower oil were from Fat Plant “Kruszwica”, Kruszwica, Poland. The potato starch was modified with different concentration of edible oil (3%, 6%, 9%) in the presence of a constant catalyst content (3%, K_2_CO_3_, Avantor Performance Materials Poland SA, Wrocław, Poland) (Table 6).

The extrusion process was performed using an Evolum EV-25 extruder (Clextral SAS, Firminy, France) with the following parameters: temperature profile (100/100/100/75/75/60 °C), throughput 7 kg/h, moisture 22%, screw speed 200 rpm, and nozzle diameter 3.5 mm. After extrusion, the samples were crushed and sieved through a 0.63 μm mesh sieve. Detailed information is presented in our previous article [19].

#### 3.1.2. Preparation of Extruded Potato Starch Films With and Without Edible Oil

In order to determine the optimal concentration of film-forming solutions, preliminary tests were conducted at extruded starch concentrations of 2.0%, 4.0%, 6.0%, 8.0%, and 10%, on a dry weight basis. Based on visual observations, 8.0% was selected as the optimal concentration for homogeneous spreading. The further experimental steps were as follows: a weighed portion (8 g, d.m.) was quantitatively transferred into 100 mL volumetric flasks, diluted to volume with distilled water, and homogenized (3 min; 10,000 rpm). To obtain films adapted to the declared research aim, the Alimi methodology [9] was used. The film-forming solutions were poured out on to a Petri dish of known and repeatable surface, in an amount corresponding to 0.15 g/cm^2^, and then dried at room temperature (20 °C) for 72 h at a relative humidity of approximately 45%.

### 3.2. Methods

#### 3.2.1. Colour Determination in CIE Lab* System

Colour was determined using the NH310 Colourimeter (Shenzhen ThreeNH Technology Co., Ltd., Guangzhou, China) in the L*, a*, and b* system for film samples in 10 repetitions. The analysis included the components L* (lightness), a* (red–green coordinate), and b* (yellow–blue coordinate). The results were averaged, and the standard deviation was calculated. Additionally, based on the obtained results, the total colour difference (Δ*E*) relative to the reference samples (C) was calculated according to Equation (1):

(1) $$∆E=\sqrt{({∆L)}^{2}+({∆a)}^{2}+{\left(∆b\right)}^{2}}$$

In the above equation, the following were used:

Δ*E*—0–1—undetectable differences;

Δ*E*—1–2—minor differences;

Δ*E*—2–3.5—medium, detectable differences;

Δ*E*—3.5–5—distinct differences;

Δ*E*—above 5 means significant differences in the hue of the colour.

The transmittance (T, %) in the Vis range was determined using a spectrophotometer (Jasco V-630, Tokyo, Japan) over a wavelength range of 380–800 nm.

#### 3.2.2. Thickness, Moisture, and Solubility Determination

The film thickness was measured using a Vernier calliper (Dasqua, Cornegliano Laudense, Italy) with an accuracy of 0.001 mm. Measurements were performed in 20 repetitions, with the sample repositioned each time to ensure that measurements were taken at different locations.

The moisture content of the films was determined for whole samples using a moisture analyzer (RADWAG WPS 50SX, Radom, Poland) in 10 repetitions. A standard drying profile and temperature of 105 °C were applied.

The obtained films were weighed (*m*_0_) with an accuracy of 0.0001 g. Samples were then quantitatively transferred to flasks, and 50 mL of distilled water was added. The resulting system was shaken for 24 h at 25 °C. Then, solutions were filtered through pre-dried (100 °C for 24 h) and pre-weighed filter papers with an accuracy of 0.0001 g. The filter papers containing the insoluble film fraction were dried to a constant weight (100 °C for 24 h). The dried filter papers were reweighed with an accuracy of 0.0001 g [19].

Solubility was calculated based on the average of two measurements using Equation (2):

(2) $$R=\;\frac{m_{0}-m_{24}}{m_{0}}\cdot 100\%\;$$

In the above, the following parameters were used:

*R*—solubility (%);

*m*_0_—initial dry mass of the sample (g);

*m*_24_—dry mass of the film after 24 h of dissolution in water (g).

All measurements were performed in triplicate.

#### 3.2.3. Hardness and Deformation Determination

The film’s strength was quantified using a Texture Analyzer AMETEK CTX (Brookfields, Toronto, ON, USA) through a penetration test using a cylindrical probe. A force was applied to the film sample to deform it to its fracture point. The film samples were cut into 60 mm diameter discs and mounted on a special table. A 3 mm diameter cylindrical probe (TA 42) was moved perpendicularly to the film surface at a constant speed of 1.0 mm/s until the probe penetrated the film (fracture point) to a depth of 8 mm. The hardness of the sample was recorded by the apparatus software (Texture Pro, 1.0.19) as the maximum force of the strain gauge (25 kg) at a specific distance (mm) necessary to obtain the desired deformation.

#### 3.2.4. FTIR

Infrared spectra of films prepared from starch extrudates, both with and without the addition of edible oils, were recorded using an FTIR spectrometer (model ALPHA II, Bruker Optics Inc., Billerica, MA, USA). The measurements utilized the attenuated total reflection (ATR) technique on a diamond crystal. The ATR method allowed direct analysis of the films with minimal preparation by placing the samples directly on a diamond crystal. Spectra were collected over a frequency range from 400 cm^−1^ to 4000 cm^−1^ in a 26-scan interferogram with a resolution of 4 cm^−1^. The FTIR spectrophotometer was controlled by OPUS 8.5 SP1 software, which was used for graphical and mathematical recording of the spectra and their processing.

#### 3.2.5. Profilometric Analysis

The roughness and topography of the extruded starch films with and without increasing oil concentrations (rapeseed or sunflower) were investigated using an optical profilometer (Contour GT Bruker, Karlsruhe, Germany). This method is optimal for 3D imaging of a wide range of surfaces, both large and small, and has the ability to perform numerical roughness analysis and plot the surface profile at any point.

Measurements were performed in six different sites for each film tested. The topography accuracy of such profilometric images is high, from a sub-nanometre up to 10 mm. The surface topography was quantified by well-known parameters: average roughness ( $R_{a}$ ), root-mean-squared roughness ( $R_{q}$ ), and peak-to-valley difference ( $R_{t}$ ). The topography parameters were analyzed by means of WS × M 5.0 (Develop 2.2 Scanning Probe Microscope Software).

#### 3.2.6. Contact Angle Measurements

The wettability of starch extrudate-based films was characterized by measuring the advancing and receding contact angles of water (Milli-Q Plus system, Merck KGaA, Darmstadt, Germany), formamide (98%, Aldrich, St. Louis, MO, USA), and diiodomethane (99%, Aldrich, St. Louis, MO, USA) using a GBX Contact Angle Metre (GBX Scientific, Romans-sur-Isère, France) equipped with an automatic drop deposition system and a camera. The measurements were carried out in a closed thermostated chamber using the sessile drop method on previously dried films.

Three probe liquids of different chemical natures (water, formamide, and diiodomethane) were used for wettability tests. In these measurements, test water acts as the primary polar reference, characterized by high surface tension (72.8 mN/m) and dominated by a strong acid–base component. The second polar component, formamide, possesses a lower surface tension (58.0 mN/m) and different donor-acceptor capabilities compared to water. The third probe liquid, diiodomethane, is a purely dispersive (non-polar) liquid with a surface tension of 50.8 mN/m. It does not have the ability to form hydrogen bonds, so contact angles are regulated almost exclusively by Lifshitz–van der Waals forces. More detailed data are presented in Table 7.

To determine the advancing contact angle, a 6 μL droplet of the probe liquid was deposited on the extruded starch film surface, while the receding contact angle was measured after sucking 2 μL of the liquid back into the syringe. In both cases, the contact angles were recorded once the droplet front had stopped moving. The GBX apparatus software calculated the contact angles from the 2D droplet profile pictures, with readings taken from both the left and right sides of the droplet. The baseline (three-phase contact line) and the top of the droplet image were settled using manual mode to ensure precision. The measurements were carried out in three independent series. For each series, a minimum of ten drops of each liquid were deposited onto each film. Each measurement was typically completed within a few seconds, a relatively short timeframe that allowed for overcoming any unfavourable reorganization of the film structure during contact with the probe liquids.

#### 3.2.7. Surface Free Energy Determination

Since the strength of solid/liquid interactions (extruded starch film/probe liquid) cannot be measured directly, their magnitude is typically estimated through the evaluation of surface free energy ( $\gamma_{S}$ ) using various theoretical approaches. In this study, we employed the Chibowski model [29,30,31], which provides a quantitative description of interactions based on contact angle hysteresis (CAH). This model estimates the surface free energy ( $\gamma_{S}$ ) using only one probe liquid by considering three variables: the surface tension of the liquid ( $\gamma_{L}$ ), the advancing contact angle ( $\theta_{a}$ ), and the receding contact angle ( $\theta_{r}$ ). This interpretation assumes that a liquid film remains behind the drop during the recession of the three-phase contact line. However, it is important to note that the resulting $\gamma_{S}$ values are apparent, as the strength of interactions, including Lifshitz–van der Waals forces and hydrogen bonds, depends on the specific liquid used and may vary from the solid side (film surface) as well Equation (3):

(3) $$\gamma_{S}=\frac{\gamma_{L}{\left(1+cos\theta_{a}\right)}^{2}}{2+{cos\theta }_{a}+cos\theta_{r}}$$

#### 3.2.8. Statistical Analysis

For the methodologies described in Section 2.1, the results were subjected to statistical analysis in the PQStat programme (version 1.8.6, PQStat Software, Poznań, Poland). One-way analysis of variance (ANOVA) was employed to compare the data, followed by Tukey’s post hoc test to determine the significance of differences between the group mean values. Statistical hypotheses were verified at the significance level of *p* < 0.05. The letters a, b, and c indicate statistically significant differences between the study groups in post hoc tests.

## 4. Conclusions

The objective of this work was to determine the surface properties of polysaccharide films, made from extruded starch dispersions, as a function of oil type and concentration, with the aim of monitoring their wettability, biocompatibility, degree of usability, and functionality. FTIR analysis, surface morphology, and roughness studies conducted by optical profilometry confirmed the contact angle results for polar liquids (water and formamide) and non-polar diiodomethane, as well as surface free energy considerations. The integration of additional methods (determination of colour, transparency, thickness, moisture, solubility, hardness, and degree of deformation) further enhanced the characterization of the polysaccharide films, potentially leading to the attainment of desirable stability and optimal wettability. Collectively, these studies demonstrate that extruded starch films hold strong potential for applications in the pharmaceutical, paper, packaging, and food industries, particularly for extending product freshness and providing resistance to oxidation and spoilage during long-term storage.

Extrudates with 6% and 9% edible oil additions were characterized by high stability and a slight increase in the structural ordering, as proved by FTIR analysis. Among them, the samples enriched with 6% sunflower oil appeared to be the most promising due to their higher brightness, medium solubility, and optimal medium permeability to Vis radiation. Additionally, they showed an average hardness slightly lower than that of the reference sample (C), but what is important from an application point of view is the lower degree of deformation. Wettability studies confirmed the structural model of starch extrudates suggested in our earlier studies. The R-series films exhibited a continuous, concentration-dependent reduction in contact angles of polar liquids, which was caused by an increase in the density of exposed ester groups and/or the polar groups of free lipids. In contrast, the S-series layers reached a minimum contact angle at 6% oil enrichemnt (S6). A further slight increase for the S9 film suggested a state of supersaturation, thereby indicating the maximum potential for practical applications. This non-linear trend, closely dependent on the oil type, perfectly aligned with the optical profilometry data. These alterations in surface roughness and wettability are directly driven by the distinct fatty acid profiles of the two edible oils, which govern the spatial packing and conformation of the grafted ester chains. Additionally, their aliphatic hydrocarbon tails significantly enhanced the dispersive intermolecular interactions with diiodomethane. This clearly revealed a strengthened lipophilic surface character of the oil-modified extrudates while maintaining the hydrophilic nature of the starch matrix.

Furthermore, wettability parameters and surface free energy analysis of the extruded starch films showed a high correlation with their functional parameters, demonstrating that the selected methods are effective tools for controlling film properties. They also provide valuable guidance in the design and qualitative/quantitative characterization of not only food coatings but also films under various environmental conditions. By utilizing modification strategies, the optimization of the surface free energy and the resulting dual hydrophilic-lipophilic character of the edible oil-modified starch extrudates enable material compatibility with diverse matrices, making such films highly valuable candidates for advanced functional applications.

## Figures and Tables

**Table 1:** Colours marked in the system L*a*b* for extruded starch films with and without (C) increasing oil concentrations (R: rapeseed oil, S: sunflower oil, 3% R3 or S3, 6% R6 or S6, and 9% R9 or S9).

Extruded Starch Film	L*	a*	b*	Δ*E*
C	83.960 ± 0.004	1.337 ± 0.011	5.556 ± 0.013	-
R3	47.055 ± 0.142	10.512 ± 0.012	31.161 ± 0.018	45.84
R6	55.636 ± 0.405	11.433 ± 0.634	33.994 ± 0.336	41.39
R9	44.074 ± 0.043	11.507 ± 0.030	33.709 ± 0.027	49.87
S3	49.725 ± 0.005	8.725 ± 0.021	25.903 ± 0.034	40.50
S6	53.865 ± 0.037	10.851 ± 0.039	31.033 ± 0.024	40.56
S9	51.017 ± 0.033	12.013 ± 0.038	32.686 ± 0.027	43.99

±—Standard deviation, L* (lightness), a* (red–green coordinate), and b* (yellow–blue coordinate). Δ*E*—total colour difference.

**Table 2:** Thickness, moisture, and solubility of extruded starch films with and without (C) increasing oil concentrations (R: rapeseed oil, S: sunflower oil, 3% R3 or S3, 6% R6 or S6, and 9% R9 or S9).

Extruded Starch Film	Thickness [mm]	Moisture [%]	Solubility [%]
C	0.133 ± 0.058 ^a^	13.355 ± 0.859 ^c^	64.130 ± 0.383 ^c^
R3	0.167 ± 0.115 ^b^	11.320 ± 0.099 ^b^	55.016 ± 2.634 ^b^
R6	0.267 ± 0.058 ^c^	10.400 ± 2.206 ^a^	56.233 ± 3.864 ^b^
R9	0.200 ± 0.100 ^bc^	10.890 ± 1.457 ^a^	60.954 ± 3.033 ^c^
S3	0.200 ± 0.100 ^bc^	12.905 ± 0.629 ^c^	57.142 ± 1.711 ^bc^
S6	0.267 ± 0.115 ^c^	12.425 ± 1.973 ^bc^	52.293 ± 1.132 ^ab^
S9	0.167 ± 0.058 ^b^	10.960 ± 0.071 ^ab^	45.739 ± 0.728 ^a^

The same letters in columns indicate values that are not significantly different at *p* = 0.05; ±—standard deviation.

**Table 3:** Hardness and deformation of extruded starch films with and without (C) increasing oil concentrations (R: rapeseed oil, S: sunflower oil, 3% R3 or S3, 6% R6 or S6, and 9% R9 or S9).

Extruded Starch Film	Hardness [g]	Deformation [mm]
C	122.00 ± 4.24 ^c^	0.775 ± 0.049 ^b^
R3	51.50 ± 6.36 ^a^	0.490 ± 0.156 ^a^
R6	46.00 ± 5.66 ^a^	0.555 ± 0.056 ^c^
R9	88.50 ± 3.54 ^b^	0.585 ± 0.021 ^a^
S3	150.50 ± 3.54 ^c^	0.670 ± 0.354 ^ab^
S6	114.00 ± 2.83 ^bc^	0.550 ± 0.113 ^a^
S9	69.00 ± 19.80 ^ab^	0.775 ± 0.021 ^b^

The same letters in columns indicate values that are not significantly different at *p* = 0.05; ±—standard deviation.

**Table 4:** Summary of major infrared bands in extruded starch films with and without (C) increasing oil concentrations (R: rapeseed oil, S: sunflower oil, 3% R3 or S3, 6% R6 or S6, 9% R9 or S9).

Range *v* (cm^−1^)	2850–2950 cm^−1^		1450–1470 cm^−1^		1735–1750 cm^−1^		1100–1250 cm^−1^		Changes in Crystallinity
Type of Vibration	C–H *		CH_2_/CH_3_ **		C=O ***		C–O–C ****		1047/1022
	*v* [cm^−1^]	Intensity Ratio	*v* [cm^−1^]	Intensity Ratio	*v* [cm^−1^]	Intensity Ratio	*v* [cm^−1^]	Intensity Ratio	
C	2858 2925	- 0.006	-	-	-	-	1146	0.020	0.7386
R3	2858 2925	- 0.009	-	-	-	-	1147	0.022	0.7445
R6	2858 2924	0.010 0.033	1452	0.027	1742	0.012	1147	0.057	0.7445
R9	2858 2923	0.028 0.044	1452	0.028	1742	0.020	1147	0.066	0.7409
S3	2858 2923	0.041 0.016	1452	0.048	1742	0.023	1147	0.106	1.0241
S6	2858 2925	0.056 0.028	1452	0.021	1742	0.011	1146	0.062	0.7381
S9	2858 2925	0.214 0.037	1452	0.024	1742	0.014	1148	0.065	0.6967

*v*—Wavenumber; * C–H stretching, typical bands for –CH_2_– and –CH_3_ groups in aliphatic chains (often associated with fatty acid esters); ** CH_2_/CH_3_—deformation of aliphatic chains in the ester structure; *** C=O stretching in the presence of the carbonyl group of the ester; **** C–O–C stretching C–O vibrations in the bond.

**Table 5:** Topographical parameters characterizing the surface topography of extruded starch films with and without (C) increasing oil concentrations (R: rapeseed oil, S: sunflower oil, 3% R3 or S3, 6% R6 or S6, and 9% R9 or S9).

Extruded Starch Film	$R_{a}$ [nm]	$R_{q}$ [nm]	$R_{t}$ [nm]
C	2.40 ± 0.42	3.09 ± 0.52	31.78 ± 5.40
R3	2.88 ± 0.53	3.62 ± 0.56	32.31 ± 6.89
R6	3.13 ± 0.61	3.99 ± 0.80	39.30 ± 11.45
R9	3.41 ± 0.41	4.26 ± 0.53	41.64 ± 13.99
S3	1.33 ± 0.16	1.72 ± 0.16	19.13 ± 6.04
S6	2.63 ± 0.62	3.41 ± 0.92	35.93 ± 18.08
S9	2.41 ± 0.39	3.07 ± 0.51	36.26 ± 12.67

$R_{a}$—arithmetical mean deviation; $R_{q}$ —root mean square roughness; $R_{t}$ —total height of the profile.

**Table 6:** The qualitative and quantitative characteristics of extruded potato starch with and without edible oil.

Code	C	R3	R6	R9	S3	S6	S9
Weight Composition [g/100 g]							
Potato starch	100	94	91	88	94	91	88
Rapeseed oil	0	3	6	9	-	-	-
Sunflower oil	0	-	-	-	3	6	9
Catalyst	-	3	3	3	3	3	3

**Table 7:** Surface tension and its components for probe liquids in mN/m.

Probe Liquid	$\gamma_{L}$	$\gamma_{L}^{LW}$	$\gamma_{L}^{+}$	$\gamma_{L}^{-}$	$\gamma_{L}^{AB}$
Water (W)	72.8	21.8	25.5	25.5	51.0
Formamide (F)	58.0	39.0	2.28	39.6	19.0
Diiodomethane (D)	50.8	50.8	0.0	0.0	0.0

## Data Availability

The original contributions presented in this study are included in the article material. Further inquiries can be directed to the corresponding author(s).
